# Characterisation of recombinant pyranose oxidase from the cultivated mycorrhizal basidiomycete *Lyophyllum shimeji *(hon-shimeji)

**DOI:** 10.1186/1475-2859-9-57

**Published:** 2010-07-14

**Authors:** Clara Salaheddin, Yoshimitsu Takakura, Masako Tsunashima, Barbara Stranzinger, Oliver Spadiut, Montarop Yamabhai, Clemens K Peterbauer, Dietmar Haltrich

**Affiliations:** 1 Food Biotechnology Laboratory, Department of Food Science and Technology, BOKU University of Natural Resources and Life Sciences, Vienna, Austria; 2 Research Centre Applied Biocatalysis, Graz, Austria; 3 Plant Innovation Center, Japan Tobacco, Iwata, Shizuoka, Japan; 4 School of Biotechnology, Suranaree University of Technology, Nakhon Ratchasima, Thailand

## Abstract

**Background:**

The flavin-dependent enzyme pyranose 2-oxidase (P2Ox) has gained increased attention during the last years because of a number of attractive applications for this enzyme. P2Ox is a unique biocatalyst with high potential for biotransformations of carbohydrates and in synthetic carbohydrate chemistry. Recently, it was shown that P2Ox is useful as bioelement in biofuel cells, replacing glucose oxidase (GOx), which traditionally is used in these applications. P2Ox offers several advantages over GOx for this application, e.g., its much broader substrate specificity. Because of this renewed interest in P2Ox, knowledge on novel pyranose oxidases isolated from organisms other than white-rot fungi, which represent the traditional source of this enzyme, is of importance, as these novel enzymes might differ in their biochemical and physical properties.

**Results:**

We isolated and over-expressed the *p2ox *gene encoding P2Ox from the ectomycorrhizal fungus *Lyophyllum shimeji*. The *p2ox *cDNA was inserted into the bacterial expression vector pET21a(+) and successfully expressed in *E. coli *Rosetta 2. We obtained active, flavinylated recombinant P2Ox in yields of approximately 130 mg per L of medium. The enzyme was purified by a two-step procedure based on anion exchange chromatography and preparative native PAGE, yielding an apparently homogenous enzyme preparation with a specific activity of 1.92 U/mg (using glucose and air oxygen as the substrates). Recombinant P2Ox from *L. shimeji *was characterized in some detail with respect to its physical and catalytic properties, and compared to the well-characterised enzymes from *Phanerochaete chrysosporium *and *Trametes multicolor*.

**Conclusion:**

*L. shimeji *P2Ox shows properties that are comparable to those of P2Ox from white-rot fungal origin, and is in general characterised by lower K_m _and *k*_cat _values both for electron donor (sugar) as well as electron acceptor (ferrocenium ion, 1,4-benzoquinone, 2,6-dichloroindophenol). While *L. shimeji *P2Ox is the least thermostable of these three enzymes (melting temperature *T*_m _of 54.9°C; half-life time of activity τ_1/2 _of 0.12 at 50°C and pH 6.5), *P. chrysosporium *P2Ox showed remarkable thermostability with *T*_m _of 75.4°C and τ_1/2 _of 96 h under identical conditions.

## Background

Pyranose 2-oxidase (P2Ox; pyranose:oxygen 2-oxidoreductase; EC 1.1.3.10) is a member of the glucose-methanol-choline (GMC) oxidoreductase family of flavin adenine dinucleotide (FAD)-dependent sugar oxidoreductases [1]. Typically, it is a homotetrameric enzyme of ~265-270 kDa [1] that is frequently found in wood-degrading basidiomycetes [2-4]. P2Ox is located in the periplasmic space and released extracellularly in later stages of development of the fungal cultures [5,6]. The enzyme was first isolated from *Polyporus obtusus *[7], and since then from several other, mainly lignocellulose-degrading, white-rot or brown-rot basidiomycetes including *Phanerochaete chrysosporium *[8,9], *Trametes *(*Coriolus*) *versicolor *[10], *Phlebiopsis gigantea *[11], *Pleurotus ostreatus *[12], *Tricholoma matsutake *[13], *Gloeophyllum trabeum *[14], and *Trametes multicolor *(synonym *T. ochracea*), which is maybe the best studied pyranose oxidase to date [1,15-17].

The reaction catalyzed by P2Ox is of the Ping Pong Bi Bi type typically found in flavoprotein oxidoreductases [18,19], and can be divided into two half reactions. In the first half reaction, the reductive half-reaction, an aldopyranose substrate reduces the flavin adenine dinucleotide (FAD) cofactor to yield FADH_2 _and 2-dehydroaldose (2-ketoaldose) as the result of oxidation of the sugar at position C-2 (Reaction 1). The second, ensuing half reaction, the oxidative half-reaction, involves re-oxidation of FADH_2 _by an electron acceptor such as oxygen (Reaction 2). During this oxidative half-reaction, a C-4a-hydroperoxyflavin intermediate is formed when oxygen is used, which is the first evidence of such an intermediate for a flavoprotein oxidase [19-21]. In addition, alternative electron acceptors such as benzoquinone can be used by pyranose oxidase instead of oxygen in the oxidative half-reaction (Reaction 3).

(1) $$\text{FAD}+\text{aldopyranose}\to {\text{FADH}}_{2}+2\text{-keto-aldopyranose}$$

(2) $${\text{FADH}}_{2}+{\text{O}}_{2}\to \text{FAD}+{\text{H}}_{2}{\text{O}}_{2}$$

(3) $${\text{FADH}}_{2}+\text{benzoquinone}\to \text{FAD}+\text{hydroquinone}$$

It has been proposed that one possible function of P2Ox could be the formation of hydrogen peroxide and thus the provision of this compound for peroxidases involved in lignin degradation. Pyranose oxidase could also function as an antimicrobial agent through its formation of H_2_O_2 _as has been proposed for the arbuscular mycorrhizal fungus *Tricholoma matsutake *[13]. As mentioned above, P2Ox can use various other electron acceptors including quinones, complexed metal ions and radicals [9,17]. Some of these alternative electron acceptors are better substrates for the enzyme than oxygen as judged from the catalytic efficiency, suggesting that P2Ox may play a role in the reduction of quinones during the process of ligninolysis [22], but this has not been studied in any detail yet. This excellent reactivity of P2Ox with alternative electron acceptors and a range of sugar substrates can be employed in various attractive applications. One possible field of application is as a bio-element in sensors and biofuel cells, where it could replace glucose oxidase, which is typically used but shows certain disadvantages. In these applications, the enzyme communicates with an electrode through small redox-active compounds or redox mediators, in a process referred to as mediated electron transfer (MET). Recently, it was shown that P2Ox can be electrically wired to graphite electrodes through the use of osmium redox polymers [23], ferrocenes or benzoquinone [24] - molecules that also serve as electron acceptors for P2Ox. These mediators transfer electrons from the enzyme to the electrode thus allowing P2Ox to be used in biosensors or biofuel cells. P2Ox is also a biocatalyst with high potential for biotransformations of carbohydrates; applications in synthetic carbohydrate chemistry, clinical analytics and in bioprocesses have been reviewed [25]. Because of the applied interest in this oxidoreductase, knowledge on P2Ox from other sources than the traditional wood-degrading fungi is of interest. P2Ox from *Lyophyllum shimeji *(*Ls*P2Ox) has been described as an antimicrobial protein effective against the rice blast fungus *Magnaporthe grisea *and sheath blight fungus *Rhizoctonia solani *in a recent patent [26], but has not been studied in detail to date. *Lyophyllum shimeji *is a mycorrhizal fungus, which grows in association with Japanese red pine and oak trees, and is cultivated commercially in Japan where it is known as 'hon-shimeji'.

In the present study, we report the heterologous expression of pyranose oxidase from *Lyophyllum shimeji *(*Ls*P2Ox) under the control of the T7 promotor in *E. coli *(Rosetta 2), and for the first time a detailed characterization of P2Ox from this mycorrhizal fungus. In addition, *Ls*P2Ox is compared with respect to some of its biochemical properties important for various applied aspects, including its kinetic properties and stability, of this enzyme to two other well-studied pyranose oxidases from lignocellulose-degrading fungi, namely *Tm*P2Ox from *T. multicolor *[17] and *Pc*P2Ox from *P. chrysosporium *[9].

## Results and Discussion

### Heterologous expression of *Ls*P2Ox-encoding cDNA

In a previous study cloning and heterologous expression of *Ls*P2Ox in *E. coli*, using the pQE30 vector (Qiagen) under control of a T5 expression system, was reported by us [26]. However, expression levels given were low (34 U/L), as apparently a significant fraction of the recombinant protein accumulated in inclusion bodies. In order to improve the expression of active *Ls*P2Ox, full-length cDNA encoding *Ls*P2Ox was expressed in *E. coli *Rosetta 2 using a pET21a(+) expression vector. The nucleotide sequence of the *p2ox *cDNA contains an ORF of 1,857 bp encoding a polypeptide of 619 amino acids. Two primers, based on the cDNA sequence and containing restriction sites for the in-frame ligation into the pET21a(+) vector were designed, and used to re-amplify the cDNA and construct the expression vector with the *p2ox *cDNA under control of the lactose-inducible T7 promoter. The resulting vector was transformed into *E. coli *Rosetta 2, and ampicillin- and chloramphenicol-resistant clones were tested for P2Ox activity after induction of *Ls*P2Ox expression. The clone with the highest activity was selected for further studies; when using this bacterial clone in small-scale expression experiments a maximal volumetric activity of 250 U/L was obtained after 68 h of cultivation in shaken flasks at an A_600 _of ~10. This corresponds to a value of approx. 130 mg of active, soluble recombinant *Ls*P2Ox per L medium as calculated from a specific activity of 1.92 U/mg of the homogenous enzyme. These fermentation yields compare well to the expression levels of other fungal P2Ox in *E. coli*. Typical yields for recombinant P2Ox production under comparable conditions range from 50-100 mg/L for P2Ox from *Trametes *spp. [27,28] to 270 mg/L for *Pc*P2Ox [9]. Furthermore, the value of 250 U/L for *Ls*P2Ox is significantly higher than the previously reported value of 34 U/L obtained with a different expression system [26]. It should also be mentioned that when using the expression host *E. coli *BL21(DE3), which we routinely use for the expression of P2Ox of different basidiomycete origin [9,29,30], results for active recombinant *Ls*P2Ox were significantly lower (data not given). *E. coli *Rosetta 2 is designed to enhance the expression of eukaryotic proteins containing codons rarely used in *E. coli *by supplying tRNAs for the codons AUA, AGG, AGA, CUA, CCC, GGA and CGG on an additional plasmid together with the chloramphenicol resistance marker. When analyzing the codons occurring in the gene encoding *Ls*P2Ox and comparison with the genes coding for *Tm*P2Ox and *Pc*P2Ox we found indeed that the above-mentioned codons occur more frequently in the *p2ox *gene of *L. shimeji *(Table 1).

**Table 1:** Comparison of codon usage frequency for selected codons that are specifically supported by *E. coli *Rosetta 2 in the genes encoding pyranose oxidase from *L. shimeji, T. multicolor *and *P. chrysosporium*.

Codon	Amino acid	Frequency of codon used (per 1000 codons)		
		***Ls*P2Ox**	***Tm*P2Ox**	***Pc*P2Ox**

AGG	Arg	11.31	4.81	4.82
AGA	Arg	4.85	0.00	1.61
CGG	Arg	8.08	4.81	1.61
GGA	Gly	25.85	14.42	8.04
AUA	Ile	3.23	1.60	1.61
CUA	Leu	6.46	1.60	0.00
CCC	Pro	19.39	16.03	32.15

The number of occurrence of the selected codons per 1000 codons is given http://www.ebioinfogen.com/biotools/codon-usage.htm.

### Enzyme purification

**Figure 1:**
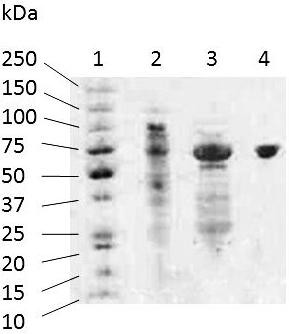
**SDS-PAGE analysis of recombinant pyranose oxidase from *Lyophyllum shimeji***. Lane 1, molecular mass marker (Precision Plus Protein Dual Color, Bio-Rad); lane 2, crude cell extract; lane 3, flow-through from AIEX column; lane 4, enzyme preparation after preparative native PAGE.

The *p2ox *cDNA was fused in frame with a C-terminal His_6_-tag encoded by the expression vector. However, IMAC could not be used for purification since the bound recombinant protein could not be eluted from the chromatographic material even with 1 M imidazole. Therefore, a two-step purification procedure based on anion exchange chromatography and preparative native PAGE was established (Table 2). Overall, *Ls*P2Ox was purified 18.8-fold from the crude cell extract to a specific activity of 1.92 U/mg. The purification procedure yielded a bright yellow enzyme preparation that was apparently homogenous as judged by SDS-PAGE (Figure 1). As estimated from this PAGE, the *Ls*P2Ox subunit has a molecular mass of ~68 kDa, which corresponds very well with the calculated theoretical mass of 68,488 Da. *Tm*P2Ox and *Pc*P2Ox were purified to apparent homogeneity via their His_6_-tag by using IMAC as previously published [9,31]. Interestingly, the construction of the C-terminal His_6_-tag was similar for the three enzymes compared, i.e., no extra spacer was added. Nevertheless, the purification of *Ls*P2Ox via IMAC was not successful when using a similar protocol successfully applied for the His-tagged proteins *Pc*P2Ox and *Tm*P2Ox. Based on the sequences of these three enzymes, a rational explanation for this behaviour is not possible. Since the protein also bound irreversibly to other standard chromatographic material used (data not shown), the reason could be through non-specific interactions of unidentified surface areas of the protein rather than the His_6_-tag.

**Table 2:** Purification of pyranose oxidase from *Lyophyllum shimeji*.

Purification step	specific activity [U/mg]	purification factor [fold]
Crude extract	0.102	1
AIEX	0.727	7.1
Native PAGE	1.92	18.8

### Kinetic properties

An attractive feature of P2Ox for various applications is its broad substrate specificity, both with respect to the electron donor and electron acceptor substrates. Therefore, the kinetic properties of *Ls*P2Ox (Michaelis constant K_m_, catalytic constant *k*_cat_, specificity constant *k*_cat_/K_m_) were determined for a range of different sugars and electron acceptors, and compared to the corresponding values determined for *Tm*P2Ox and *Pc*P2Ox. These latter two enzymes are both derived from lignocellulose-degrading basidiomycetes, and are among the best-studied pyranose oxidases to date. Kinetic constants for a range of electron donor substrates - various monosaccharides and the disaccharide melibiose - determined in the presence of air oxygen as saturating electron acceptor are summarised in Table 3. In general, *Ls*P2Ox is characterised by lower Michaelis constants as compared to those determined for *Tm*P2Ox and *Pc*P2Ox, albeit also by catalytic constants that are significantly lower for these sugar substrates than those measured for the other two enzymes. D-Glucose is the favoured substrate for all three enzymes as judged by the catalytic efficiencies, while the disaccharide melibiose is the poorest, mainly because of a very unfavourable Michaelis constant. This unfavourable binding may be explained by a rather narrow active site of P2Ox with the access being restricted by the active-site loop [16]. Similarly, kinetic constants were determined for *Ls*P2Ox, *Tm*P2Ox and *Pc*P2Ox for the one-electron non-proton acceptor ferrocenium ion Fc^+ ^(ferrocenium hexafluorophosphate FcPF_6_) and the two-electron proton acceptors 1,4-benzoquinone (BQ) and 2,6-dichloroindophenol (DCIP) at a saturating concentration of 100 mM D-glucose (Table 4). Again, *Ls*P2Ox shows lower K_m _as well as lower *k*_cat _values for these electron acceptors compared to *Tm*P2Ox and *Pc*P2Ox. Judged by the catalytic efficiencies, BQ is the preferred substrate for all three enzymes.

**Table 3:** Comparison of apparent kinetic constants of recombinant pyranose oxidase from *Lyophyllum shimeji *(*Ls*P2Ox), *Trametes multicolor *(*Tm*P2Ox) and *Phanerochaete chrysosporium *(*Pc*P2Ox) for various electron donor substrates.

Enzyme		K_m _[mM]	*k*_cat _[s^-1^]	*k*_cat_/K_m _[mM^-1^s^-1^]	rel. *k*_cat_/K_m_^a ^[%]
*Ls*P2Ox	D-glucose	0.314	6.92	22.1	100
	D-galactose	3.84	1.02	0.265	100
	D-xylose	6.30	5.48	0.867	100
	L-sorbose	14.7	7.70	0.524	100
	melibiose	72.8	0.839	0.0115	100

*Tm*P2Ox	D-glucose	0.698	35.4	50.8	230
	D-galactose	8.09	2.73	0.337	127
	D-xylose	22.2	17.8	0.804	92.7
	L-sorbose	35.6	31.6	0.887	169
	melibiose	759	5.25	0.00691	60.1

*Pc*P2Ox	D-glucose^b^	0.84	83.1	98.9	448
	D-galactose^b^	2.94	4.87	1.66	626
	D-xylose^b^	20.9	44.9	2.15	248
	L-sorbose^b^	23.5	58.8	2.50	477
	melibiose	124.8	2.35	0.0189	164

^a^catalytic efficiency *k*_cat_/K_m _relative to the value calculated for *Ls*P2Ox

^b^data taken from reference [9]

Kinetic data were determined at 30°C, pH 6.5 and using oxygen (air saturation) as electron acceptor.

**Table 4:** Comparison of apparent kinetic constants of recombinant pyranose oxidase from *Lyophyllum shimeji *(*Ls*P2Ox), *Trametes multicolor *(*Tm*P2Ox) and *Phanerochaete chrysosporium *(*Pc*P2Ox) for the electron acceptor substrates ferrocenium ion (Fc^+^), 1,4-benzoquinone (BQ) and 2,6-dichloroindophenol (DCIP).

Enzyme		K_m _[mM]	*k*_cat _[s^-1^]	*k*_cat_/K_m _[mM^-1^s^-1^]	rel. *k*_cat_/K_m_^a^[%]
*Ls*P2Ox	Fc^+^	0.187	39.9	213	100
	BQ	0.033	92.3	2760	100
	DCIP	0.187	67.3	361	100

*Tm*P2Ox	Fc^+^	0.507	291	574	269
	BQ	0.253	225	895	32.4
	DCIP	0.413	42.0	102	28.2

*Pc*P2Ox	Fc^+^	0.330	228	691	324
	BQ^b^	0.110	400	3640	132
	DCIP^b^	0.051	108	2120	587

^a^catalytic efficiency *k*_cat_/K_m _relative to the value calculated for *Ls*P2Ox

^b^data taken from reference [9]

Kinetic data were determined at 30°C, pH 6.5 and using D-glucose (100 mM) as saturating electron donor.

**Figure 2:**
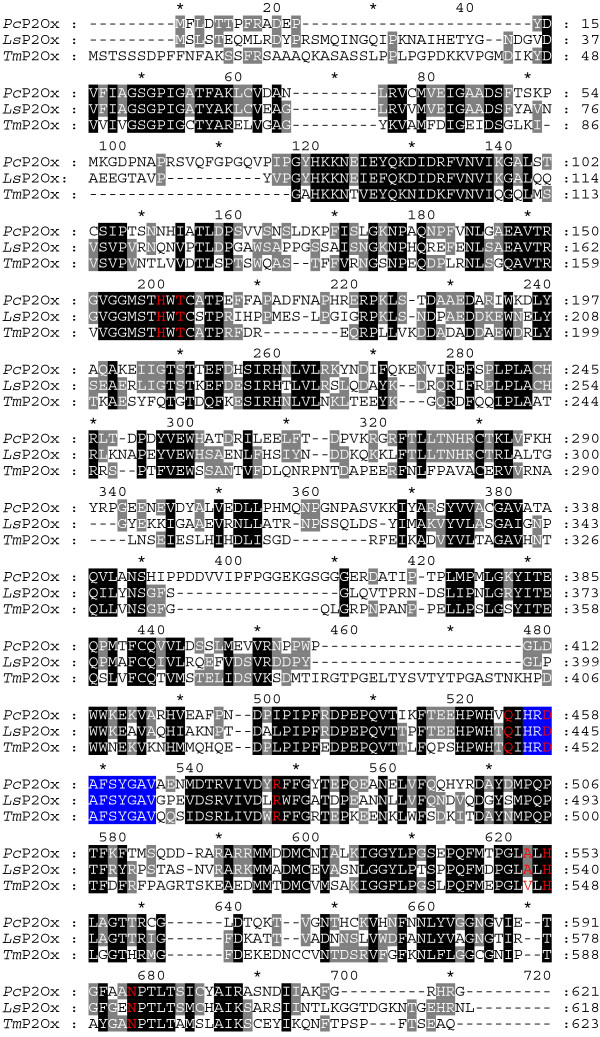
**Protein sequence alignment of pyranose oxidase from *Lyophyllum shimeji*, *Ls*P2Ox [GenBank: **BAD12079**], *Phanerochaete chrysosporium*, *Pc*P2Ox [GenBank: **AAS93628**] and *Trametes multicolor, Tm*P2Ox [GenBank: **AAX09279.1**]**. The active-site loop, which is important for substrate recognition and catalysis, is marked by a dark-blue background, while residues that are proposed to be involved in substrate binding and/or catalysis are highlighted in red. Residues that are strictly conserved in these three sequences are shaded black, while conserved residues found in two of these sequences are highlighted in grey.

The substrate specificity as well as kinetic properties of recP2Ox from *L. shimeji *described here are in overall agreement with those reported for P2Ox isolated from white-rot fungi. *Ls*P2Ox is, however, generally characterised by significantly lower catalytic constants *k*_cat _as well as by decreased Michaelis constants K_m _for most of these substrates when compared to *Tm*P2Ox and *Pc*P2Ox. Recently, we published the structure of *Tm*P2Ox ligated with a bound, poor substrate, 2-deoxy-2-fluoro-D-glucose [16]. This structural analysis allowed the identification of active-site residues that directly take part in substrate binding. These residues include (in *Tm*P2Ox numbering, corresponding positions for *Ls*P2Ox are given in brackets) Gln448 (Gln441), Asp452 (Asp445), Arg472 (Arg465), His548 (His540), and Asn593 (Asn583). As shown in a sequence alignment, these active-site residues are strictly conserved for *Ls*P2Ox, *Tm*P2Ox and *Pc*P2Ox (Figure 2). The only exception to this is found at Val546 in *Tm*P2Ox, which corresponds to Ala538 and Ala551 in *Ls*P2Ox and *Pc*P2Ox, respectively. At this position, the interaction with the sugar substrate is through the carbonyl oxygen of the polypeptide main chain, forming a hydrogen bond with the C1 hydroxyl group of the sugar substrate, rather than through the amino acid side chain; hence the replacement of one hydrophobic aliphatic amino acid with another one should neither affect this interaction nor the properties of the active site. Furthermore, it was shown that the active-site loop ^450^His-Val^459 ^is important for substrate recognition and catalysis [1,16]. This active-site loop is also strictly conserved in *Ls*P2Ox (^443^HRDAFSYGAV^452^) when compared to both *Tm*P2Ox and *Pc*P2Ox as is evident from Figure 2. In addition, the residues His548 (His540), Asn593 (Asn583), Thr169 (Thr172) and His167 (His170) were shown to be important for catalysis and covalent binding of the prosthetic group [1,16,20]. Again, these residues are conserved (Figure 2). Because of this strict conservation of important active-site residues, the differences observed in catalytic properties of *Ls*P2Ox must stem from subtle structural variations of the active site. Whether these differences are typically distinguishing P2Ox of ectomycorrhizal and white-rot fungal origin cannot be answered to date, and more pyranose oxidases from ectomycorrhizal fungi have to be studied to answer this question.

### pH dependence of activity

**Figure 3:**
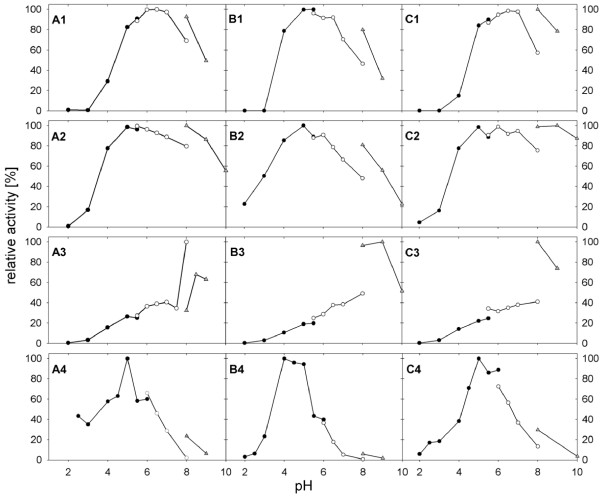
**pH-activity profiles of pyranose oxidase from *L. shimeji *(A), *T. multicolor *(B), *P. chrysosporium *(C) in the presence of the electron acceptors (1) O_2_, (2) 1,4-benzoquinone, (3) ferrocenium ion Fc^+^, and (4) 2,6-dichloroindophenol**. The buffers used were 100 mM citrate (filled square), 100 mM phosphate (open square), and 100 mM borate (open triangle).

pH/activity profiles were determined for the electron acceptors oxygen, DCIP, Fc^+ ^and BQ from pH 2.0 to 10.0 for recombinant *Ls*P2Ox, *Tm*P2Ox and *Pc*P2Ox (Figure 3). These profiles are well comparable for the three enzymes studied. The profiles for both oxygen and BQ are rather broad with bell-shaped curves, showing more than 80% activity in the range of pH 5.0 to 7.0. In contrast, activity with DCIP shows a strong dependence on the pH with a sharp optimum of 5.0 for *Ls*P2Ox and *Pc*P2Ox, and a somewhat lower optimum of 4.0-5.0 for *Tm*P2Ox. P2Ox activity almost linearly increased with increasing pH for Fc^+ ^in the range of pH 2 to 8, regardless of its source. The three enzymes furthermore showed higher activity in the presence of borate buffer, which has not been shown previously. Artolozaga et al. showed that other buffer systems used at a comparable alkaline pH range (Tris-HCl buffer, Glycine-NaOH buffer) did not show a comparable effect [8].

### Thermal stability

**Figure 4:**
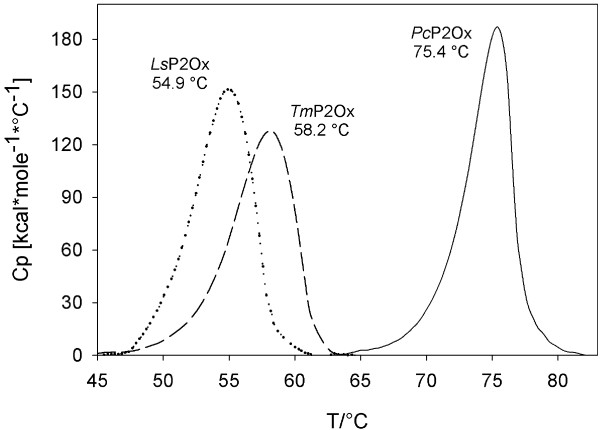
**Denaturing thermograms determined by differential scanning calorimetry of pyranose oxidase from *L. shimeji *(LsP2Ox; dotted line); *T. multicolor *(*Tm*P2Ox; dashed line), and *P. chrysosporium *(*Pc*P2Ox, solid line)**.

Pyranose oxidase from *L. shimeji *was investigated by differential scanning calorimetry and compared to the enzymes from *T. multicolor *and *P. chrysosporium *in order to study heat-induced unfolding and thus thermodynamic stability [32]. Cooperative unfolding peaks were observed in the first heating cycle (Figure 4). Samples showed significant protein precipitation after this first heating cycle, indicating irreversible aggregation, and hence no cooperative melting peaks were observed in a second heating cycle. Because of this, the thermodynamic values associated with the heat absorption data, which are calculated by equations that are based on reversible thermodynamic criteria, are only indicative. The measured values for the melting temperature *T*_m_, however, can be regarded as informative since irreversible aggregation occurs only once the unfolding process is complete, after reaching the melting point of the respective protein. These melting temperatures *T*_m _determined are 54.9°C for *Ls*P2Ox, while *Tm*P2Ox and *Pc*P2Ox have *T*_m _values of 58.2°C and 75.4°C, respectively, indicating that *Ls*P2Ox is the least thermostable of the three enzymes compared.

**Figure 5:**
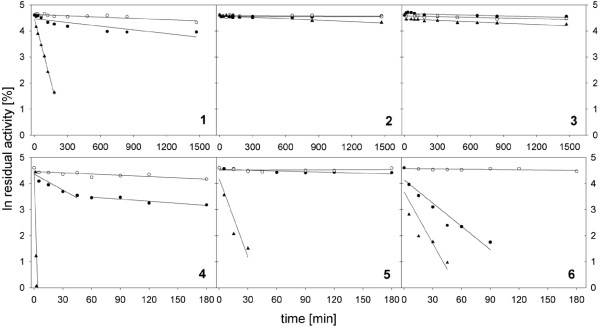
**Inactivation kinetics of pyranose oxidase from *L. shimeji *(filled triangle), *T. multicolor *(filled square) and *P. chrysosporium *(open square) at 40°C (1-3) and 50°C (4-6)**. Samples were incubated at pH 4.0 (1, 4), pH 6.5 (2, 5), and pH 8.0 (3, 6).

To further investigate thermal stability, kinetic stabilities (i.e., the length of time an enzyme remains active before undergoing irreversible inactivation; [32]) were determined at different temperatures (40 and 50°C) and pH values (pH 4.0, 6.5, 8.0) for the three P2Ox samples. Again, *Ls*P2Ox shows the least stability when compared to *Tm*P2Ox and *Pc*P2Ox (Figure 5). While these enzymes show little inactivation at 40°C and pH 6.5 and 8.0 (half-life times τ_1/2 _of approximately 60 h for *Ls*P2Ox), inactivation is more pronounced at pH 4.0, especially for *Ls*P2O which shows a half-life time τ_1/2 _of 43 min at this temperature. A more detailed analysis of the inactivation kinetics at 50°C is shown in Table 5. Again, *Pc*P2Ox proved to be the most stable of the enzymes compared, and significant differences in thermal stability were found for the different enzymes. All three enzymes were inactivated most rapidly at pH 4.0, with τ_1/2 _values ranging from 0.59 to 430 min, and were found to be most stable at pH 6.5 (τ_1/2 _values ranging from 0.12 to 96 h).

**Table 5:** Kinetic stability of recombinant pyranose oxidase from *Lyophyllum shimeji *(*Ls*P2Ox), *Trametes multicolor *(*Tm*P2Ox) and *Phanerochaete chrysosporium *(*Pc*P2Ox) determined at 50°C and various pH values

	pH 4.0		pH 6.5		pH 8.0	
			
Enzyme	inactivation constant *k*_in _[min^-1^]	half-life τ_1/2 _[min]	inactivation constant *k*_in _[min^-1^]	half-life τ_1/2 _[min]	inactivation constant *k*_*i*n _[min^-1^]	half-life τ_1/2 _[min]
*Ls*P2Ox	11800 × 10^-4^	0.587	1000 × 10^-4^	6.92	659 × 10^-4^	10.5
*Tm*P2Ox	204 × 10^-4^	33.9	98.6 × 10^-4^	70.3	297 × 10^-4^	23.3
*Pc*P2Ox	16.3 × 10^-4^	433	1.20 × 10^-4^	5760	3.85 × 10^-4^	1800

*Ls*P2Ox was found to be less thermostable than *Tm*P2Ox and *Pc*P2Ox, both when using DSC/melting temperature and inactivation kinetics as a measure for thermostability. The melting temperature *T*_m _was 3°C lower than that found for *Tm*P2Ox, while it was more than 20°C lower compared to *Pc*P2Ox. These differences are also reflected in the τ_1/2 _values, the half-life times of activity, at 50°C and pH 6.5, which are approx. 10-fold higher for *Tm*P2Ox and 800-fold higher for *Pc*P2Ox. As judged from thermostability and kinetic data, pyranose oxidase from *P. chrysosporium *seems to be the clearly better suited biocatalyst for various biotechnological applications when compared to *Ls*P2Ox and *TmP2Ox*.

## Conclusions

The enzyme pyranose oxidase has gained increased attention during the last years because of a number of attractive applications that have been proven or proposed for this enzyme. P2Ox is a unique biocatalyst for a number of uses in carbohydrate transformations, converting simple sugars or sugar derivatives into valuable intermediates that can be further used for the synthesis of rare sugars, fine chemicals or pharmaceuticals [25]. Recently, it was shown that P2Ox can be used conveniently as an anodic component in biofuel cells, replacing glucose oxidase (GOx), which traditionally is used in these applications [23,24]. P2Ox offers several advantages over GOx for this use, e.g., its much broader substrate specificity is opening the possibility of utilising additional carbohydrates for these applications. Because of this renewed interest in P2Ox, knowledge on novel pyranose oxidases isolated from organisms other than white-rot fungi, which represent the traditional source of this enzyme, is of importance, as these novel enzymes might differ in their biochemical and physical properties. Furthermore, this knowledge on a variety of pyranose oxidases and their characteristics can be used for tailoring the properties of P2Ox, e.g., by combinatorial methods of directed evolution such as DNA shuffling. In this approach of enzyme engineering, two or more genes from different sources are the starting point for the engineering work [33]. This method allows the generation of a much larger spectrum of diversity than by natural recombination or mutational mechanisms, since two or more homologues from multiple species in different ratios are used for recombination [34,35]. Recently, we could show that various approaches of semi-rational design can significantly improve the properties of *Tm*P2Ox for applications in biofuel cells, e.g. by increasing thermostability [31] or catalytic properties with respect to various electron acceptor substrates [36], making it possible to achieve a higher energy output of an enzymatic biofuel cell when using the same concentration of sugar substrate [24], and hence a combinatorial engineering approach would certainly be worthwhile for further improvements of pyranose oxidase.

In this study we report the detailed characterization of a P2Ox from the ectomycorrhizal basidiomycete *Lyophyllum shimeji*. Relatively little is known about enzyme activities of ectomycorrhiza involved in the metabolism of lignin. Genes equivalent to various peroxidases such as lignin peroxidase and manganese peroxidase as found in wood-degrading white-rot fungi appear to be widespread in ectomycorrhizal basidiomycetes [37,38], but the corresponding gene products have not been studied in detail so far. Laccase, along with a range of related phenol-oxidising activities, which is also thought to play an important role in lignin degradation, has also been reported in several ectomycorrhizal fungi [27,28,39], but again the corresponding enzymes have not been studied in detail pertaining to their biochemical properties. Hence, the study of enzymes related to ligninolysis - such as pyranose oxidase - and derived from ectomycorrhizal basidiomycetes is also of fundamental interest.

Pyranose oxidase from *L. shimeji *is very similar to P2Ox isolated from wood-degrading white-rot fungi such as *P. chrysosporium *or *T. multicolor *with respect to its physical and biochemical properties. P2Ox has been speculated to be an important source of hydrogen peroxide, which is an essential co-substrate for peroxidases. As judged from the biochemical similarities of *Ls*P2Ox with pyranose oxidases from white-rot sources, the enzyme could play a similar role in ectomycorrhizal fungi, supplying peroxidases with hydrogen peroxide.

Whether the differences observed for P2Ox from ectomycorrhizal *L. shimeji *and the white rotters *T. multicolor *and *P. Phanerochaete*, e.g. pertaining to kinetic properties or stability, are typically distinguishing P2Ox of ectomycorrhizal and white-rot fungal origin cannot be answered to date, as more pyranose oxidases from ectomycorrhizal fungi will have to be studied to answer this question unequivocally.

## Methods

### Organisms and plasmids

P2Ox from *T. multicolor *and *P. chrysosporium *were heterologously expressed in *E. coli *BL21(DE3) (Fermentas; St. Leon-Roth, Germany) using the pET21-d(+) and pET21-a(+) expression vector (Novagen; Madison, WI), respectively [9,16]. The recombinant enzymes were fused with a C-terminal His_6_-tag for one-step purification via immobilized metal affinity chromatography (IMAC). Construction of the *Ls*P2Ox cDNA in the pBluescript vector (Stratagene; La Jolla, CA) was recently reported [26]. Briefly, *L. shimeji *fruiting bodies were obtained from Shiga Forest Research Center (Yasu-shi, Japan). From these fruiting bodies a cDNA library was constructed and *Ls*P2Ox cDNA was isolated.

### Cloning of P2Ox from *Lyophyllum shimeji*

The *Ls*P2Ox cDNA sequence was amplified by PCR using two modified flanking primers based on the nucleotide sequence (GenBank accession number AB119106), and containing a *Hin*dIII and *Not*I restriction site: LSHindIII_fwd: 5'-AAGCTTATGTCTCTCTCAACCGAGCAG-3' and LSNotI_rev: 5'-GCGGCCGCAAGGTTGCGATGCTCGCCTG-3'. The amplified sequence was temporarily cloned blunt into the pJET vector using the pJET Cloning Kit (Fermentas). The gene was excised and ligated into the pET21a(+) expression vector in frame with the C-terminal His_6_-tag; this tag was added directly at the C-terminus without the insertion of an additional spacer. This plasmid was then transformed and *Ls*P2Ox heterologously expressed in *E. coli *Rosetta 2(DE3)pLacI (Novagen).

### Cultivation and expression of P2Ox

Cultivation of *E. coli *Rosetta 2 and BL21(DE3) for production of recombinant enzymes was done in TB medium (24 g/L yeast extract, 12 g/L peptone from casein, 4 mL/L glycerol, 1 M potassium phosphate buffer pH 7.5). *E. coli *Rosetta 2 (for over-expression of *Ls*P2Ox) was pre-cultivated in 25 mL of TB-medium containing 100 μg/mL ampicillin and 34 μg/mL chloramphenicol. *E. coli *BL21(DE3) (for *Pc*P2Ox, *Tm*P2Ox) was pre-grown in shaken flasks using 25 mL of TB medium and 100 μg/mL ampicillin. After 4 h of growth the cell suspensions were transferred into baffled shaken flasks containing 250 mL of the respective medium and 0.5% lactose for induction of recombinant protein expression. Cultures were incubated at 18°C, 110 rpm and 68 h for *E. coli *Rosetta 2, and 25°C, 110 rpm and 24 h for *E. coli *BL21(DE3). Cells were harvested by centrifugation for 15 min at 10,000 × *g *and 4°C, the pellets were resuspended in a threefold volume of the buffer used for the subsequent enzyme purification procedure, and cells were disrupted in a continuous homogenizer (APV Systems; Silkeborg, Denmark) after adding phenyl methyl sulfonyl fluoride (PMSF; 1 g/L) as a protease inhibitor. The homogenates were clarified by ultracentrifugation at 35,000 × *g *for 30 min at 4°C, and the recombinant proteins were purified from these crude cell extracts [40].

### Enzyme purification

Even though *Ls*P2Ox was His-tagged, it could not be purified by IMAC as it irreversibly bound onto the Ni^2+ ^column used (Ni^2+^-charged Chelating Sepharose Fast Flow; Amersham Pharmacia, Uppsala, Sweden). Hence, a two-step purification process was established for *Ls*P2Ox using anionic exchange chromatography (AIEX) and preparative native PAGE. AIEX was done on a 450-mL Q-Sepharose (Amersham Pharmacia) column pre-equilibrated with Buffer A [50 mM 2-(*N*-morpholino)ethanesulfonic acid (MES), 50 mM NaCl, pH 6.0]. After applying the crude extract to the column and washing with Buffer A, *Ls*P2Ox was collected in the flow-through while protein contamination from the crude extract bound to the chromatographic material. The active fractions of the flow-through were concentrated by ultrafiltration using an Amicon Ultra Centrifugal Filter Device with a 10-kDa cut-off membrane (Millipore; Billerica, MA), and loaded onto a preparative native gel (10% acrylamide, 0.2% ammonium persulfate, 0.2% Temed). After running the Native PAGE, *Ls*P2Ox was detected by active staining. One lane of the gel was covered with a layer of the reagent normally used in the standard chromogenic ABTS assay (see below) for the spectrophotometrical enzyme activity determination. The active protein band was identified by its green colour, and *Ls*P2Ox could be extracted from the remaining lanes with potassium-phosphate buffer (50 mM, pH 6.5). *Tm*P2Ox and *Pc*P2Ox were purified by IMAC as previously reported [40]. In short, crude extracts were passed through a 100-mL Ni^2+^-immobilized column (Chelating Sepharose Fast Flow; Amersham Pharmacia). After washing the column with Buffer B (50 mM KH_2_PO_4_, pH 6.5, 0.5 M sodium chloride) containing 20 mM imidazole and applying the respective crude extracts, protein was eluted by a linear imidazole gradient of 20 to 1000 mM imidazole in Buffer B. Active fractions were pooled and again concentrated by ultrafiltration as above.

SDS-PAGE was performed on a PhastSystem unit (Amersham Pharmacia) according to the manufacturer's instructions using precast gels (precast PhastGel, Gradient 8-25) and the Precision Plus Protein Dual Color Kit (Biorad) as molecular mass standard.

### Enzyme activity assay

P2Ox activity was measured with the standard chromogenic 2,2'-azinobis(3-ethylbenzthiazolinesulfonic acid) (ABTS) assay [3]. A sample of diluted enzyme (10 μL) was added to 980 μL of assay buffer containing horseradish peroxidase (5.72 U), ABTS (0.59 mg) and phosphate buffer (50 mM, pH 6,5). The reaction was started by adding D-glucose (20 mM). The absorbance change at 420 nm was recorded at 30°C for 180 s (ϵ_420 _= 43.2 mM^-1 ^cm^-1^). Protein concentrations were determined by the Bradford assay using the Bio-Rad Protein Assay Kit with BSA as standard.

### Steady-state kinetic measurements

Steady-state kinetic constants were measured for *Ls*P2Ox, *Tm*P2Ox and *Pc*P2Ox for different electron donor (sugar) as well as electron acceptor substrates. Unless otherwise stated, all measurements were performed at 30°C in 50 mM potassium phosphate buffer (pH 6.5). Measurements of kinetic constants for various sugar substrates were done with oxygen (air saturation) and the routine ABTS-peroxidase assay. D-Glucose, D-galactose, D-xylose, L-sorbose and melibiose were varied over a range of 0.25-500 mM. Furthermore, kinetic constants for the electron acceptors 2,6-dichloroindophenol (DCIP), ferrocenium ion Fc^+ ^(using hexafluorophosphate Fc^+^PF_6_^-^; Aldrich, Steinheim, Germany) and 1,4-benzoquinone (BQ) were determined. These experiments were performed with a constant saturating D-glucose concentration of 100 mM. DCIP was varied over a range of 7.5 to 600 μM and the time-dependent reduction was measured at 520 nm (ϵ_520 _= 6.8 mM^-1 ^cm^-1^) [41]. Fc^+ ^was varied from 5 to 600 μM and measured at 300 nm (ϵ_300 _= 4.3 mM^-1 ^cm^-1^), while BQ was varied from 10 to 750 μM and measured at 290 nm (ϵ_290 _= 2.24 mM^-1 ^cm^-1^). In short, 10 μL of appropriately diluted enzyme was added to 990 μL of buffer containing D-glucose and the electron acceptor substrate, and which had been flushed with nitrogen to remove oxygen. All the kinetic constants were calculated using non-linear least-squares regression by fitting the observed data to the Michaelis-Menten equation. Turnover numbers (*k*_cat_) were calculated using a molecular mass of 68 kDa for the P2Ox subunits.

### pH dependence of activity

pH-activity profiles of *Ls*P2Ox, *Tm*P2Ox and *Pc*P2Ox were determined for oxygen, DCIP (0.3 mM), Fc^+^ (0.3 mM) and BQ (0.5 mM) in the range of pH 2.0 to 10.0, using the buffer system sodium citrate (pH 2.0-6.0), potassium phosphate (pH 6.0-8.0) and sodium borate (pH 8.0-10.0), each at a concentration of 100 mM. Activity measurements were performed with a constant D-glucose concentration of 100 mM at 30°C.

### Thermal stability

Kinetic stability of *Ls*P2Ox, *Tm*P2Ox and *Pc*P2Ox was determined at 40 and 50°C and at pH 4.0, 6.5 and 8.0, using the same buffers as above. Samples were taken at various time points *t *and P2Ox activity (*A*) was measured using the standard ABTS assay. A thermal cycler and thin-walled PCR tubes were used for all thermostability measurements. Residual activities (*A_t_*/*A_0_*, where *A*_*t *_is the activity measured at time *t *and *A*_*0 *_is the initial activity at *t *= 0) were plotted versus the incubation time. Inactivation constants *k*_in _were obtained by linear regression of (ln activity) versus *t*. The half-life values of thermal inactivation τ_1/2 _were calculated using τ_1/2 _= ln 2/*k*_in _[32].

Thermodynamic stability was measured by determination of the melting temperatures *T*_m_, using differential scanning calorimetry (DSC) and a MicroCal VP-DSC instrument (MicroCal, Northampton, MA) in the range of 20-100°C at a scan rate of 1°C per min on 4 μM protein samples in 50 mM phosphate buffer (pH 6.5). Samples were degassed by continuous stirring for 15 min in vacuum immediately before starting the measurements. For baseline correction a buffer blank was scanned in the second chamber and subtracted. Evaluation of data was performed by the Origin 7.5 software (OriginLab Corporation, Northampton, MA).

## List of abbreviations

ABTS: 2,2'-azinobis(3-ethylbenzthiazolinesulfonic acid); BQ: 1,4-benzoquinone; DCIP: 2,6-dichloroindophenol; Fc^+^: ferrocenium ion; *Ls*P2Ox: pyranose oxidase from *Lyophyllum shimeji*; P2Ox: pyranose oxidase; *Pc*P2Ox: pyranose oxidase from *Phanerochaete chrysosporium*; *T*_m_: melting temperature; *Tm*P2Ox: pyranose oxidase from *Trametes multicolor; *τ_1/2_: half-life times

## Competing interests

The authors declare that they have no competing interests.

## Authors' contributions

CS and BS carried out the molecular biology studies as well as the biochemical characterisation experiments of the enzyme and drafted the manuscript. YT and MT participated in the molecular biology studies as well as in the sequence alignment, and revised the draft. OS checked the data and revised the draft. MY co-supervised the molecular biology studies. CKP and DH conceived of the study, and edited the manuscript. All authors read and approved the final manuscript.
